# Effects of a standard provision versus an autonomy supportive exercise referral programme on physical activity, quality of life and well-being indicators: a cluster randomised controlled trial

**DOI:** 10.1186/1479-5868-11-10

**Published:** 2014-01-29

**Authors:** Joan L Duda, Geoffrey C Williams, Nikos Ntoumanis, Amanda Daley, Frank F Eves, Nanette Mutrie, Peter C Rouse, Rekha Lodhia, Ruth V Blamey, Kate Jolly

**Affiliations:** 1 School of Sport, Exercise and Rehabilitation Sciences, University of Birmingham, Birmingham B15 2TT, UK; 2 Department of Clinical and Social Sciences in Psychology, University of Rochester, Rochester, New York 14627, USA; 3 School of Health & Population Sciences, University of Birmingham, Birmingham B15 2TT, UK; 4 School of Education, Institute for Sport, Physical Education and Health Sciences, University of Edinburgh, Edinburgh EH8 8AQ, UK

**Keywords:** Exercise on referral, Physical activity promotion, Self determination theory, Autonomy support, Autonomous motivation, Need satisfaction, Subjective vitality, Dartmouth CO-OP charts

## Abstract

**Background:**

The National Institute for Health and Clinical Excellence in the UK has recommended that the effectiveness of ongoing exercise referral schemes to promote physical activity should be examined in research trials. Recent empirical evidence in health care and physical activity promotion contexts provides a foundation for testing the feasibility and impact of a Self Determination Theory-based (SDT) exercise referral consultation.

**Methods:**

An exploratory cluster randomised controlled trial comparing standard provision exercise referral with an exercise referral intervention grounded in Self Determination Theory. Individuals (N = 347) referred to an exercise referral scheme were recruited into the trial from 13 centres.

Outcomes and processes of change measured at baseline, 3 and 6-months: Minutes of self-reported moderate or vigorous physical activity (PA) per week (primary outcome), health status, positive and negative indicators of emotional well-being, anxiety, depression, quality of life (QOL), vitality, and perceptions of autonomy support from the advisor, need satisfaction (3 and 6 months only), intentions to be active, and motivational regulations for exercise.

Blood pressure and weight were assessed at baseline and 6 months.

**Results:**

Perceptions of the autonomy support provided by the health and fitness advisor (HFA) did not differ by arm. Between group changes over the 6-months revealed significant differences for reported anxiety only. Within arm contrasts revealed significant improvements in anxiety and most of the Dartmouth CO-OP domains in the SDT arm at 6 months, which were not seen in the standard exercise referral group. A process model depicting hypothesized relationships between advisor autonomy support, need satisfaction and more autonomous motivation, enhanced well being and PA engagement at follow up was supported.

**Conclusions:**

Significant gains in physical activity and improvements in quality of life and well-being outcomes emerged in both the standard provision exercise referral and the SDT-based intervention at programme end. At 6-months, observed between arm and within intervention arm differences for indicators of emotional health, and the results of the process model, were in line with SDT. The challenges in optimising recruitment and implementation of SDT-based training in the context of health and leisure services are discussed.

**Trial registration:**

The trial is registered as Current Controlled trials ISRCTN07682833.

## Background

Reviews of the literature and meta-analyses have revealed weak and inconsistent evidence regarding the impact of participation in exercise referral schemes, when compared to usual care, on increases in physical activity, well-being, quality of life or clinical physical health outcomes (e.g., blood pressure) [1-3]. Exercise referral schemes have been defined as “clear referrals by primary care professionals to third party service providers to increase physical activity or exercise” via participants’ engagement in limited time (usually 10–12 weeks) and programmes tailored to individuals following “initial assessment and monitoring throughout” [2]. For example, a large RCT examining the impact of an exercise referral programme in relatively inactive women in New Zealand reported significantly greater improvements in the proportion of the intervention group who were physically active at 1 and 2 years than control participants and also greater improvements in quality of life, but a higher rate of falls and injuries in the intervention group [4]. Sorensen and colleagues reported no significant differences between two active exercise referral interventions, both including motivational interviewing, at the programme end or at 10 months follow-up [5].

Results are also mixed when comparisons between exercise referral schemes are made with different physical activity interventions such as walking programmes. A trial within the context of a GP exercise referral scheme [6] examined the effectiveness of three interventions (supervised exercise classes, an instructor-led walking group, and an advice only condition). Whilst levels of moderate physical activity (PA) were augmented in all 3 arms at 10 weeks, at 6 month and 1 year follow-ups only the two exercise conditions exhibited significant increases when compared to baseline, but there were no between group significant differences in duration of moderate PA or reported anxiety and depression symptoms at any of the three assessment points. Gusi and colleagues reported that a primary care-based supervised walking programme for overweight or moderately depressed women aged 60 years or greater, was highly cost-effective compared to usual care [7].

Overall then, the literature to date concerning the impact of exercise on referral schemes is limited and not particularly encouraging. As a result of the equivocal findings regarding the impact of exercise on referral programmes, the UK National Institute for Health and Clinical Excellence [8] recommended that “practitioners, policy makers, and commissioners should only endorse exercise referral schemes to promote physical activity that are part of a properly designed and controlled research study to determine effectiveness”. In part, this trial was a response to this guidance.

### Pulling from theory in exercise on referral interventions

Past work has pointed to the importance of theory in developing, implementing and evaluating interventions centred on promoting active living, well-being and quality of life [9] and it has been argued that trials examining the effectiveness of exercise referral schemes should be theory-based [2]. According to Michie and colleagues “a ‘good’ theory … will specify causal relations between variables and proffer implications for designing interventions to promote people’s health” [10]. Theoretical frameworks are also called upon to make proposals regarding the mechanisms by which interventions, such as exercise referral schemes, can impact PA adoption and maintenance as well as associated outcomes [11].

One contemporary approach that holds relevance for intervention design and promise for further understanding of the processes leading to sustained motivation and optimal functioning/well-being is self determination theory (SDT) [12,13]. SDT centres on the determinants and consequences of more autonomous (e.g. enjoyment and/or personal value) and controlled reasons (e.g. guilt and/or extrinsic reinforcements) for behavioural engagement. As a mini-theory within the SDT framework, Basic Needs Theory [14] holds that the satisfaction of the basic psychological needs of competence, autonomy and relatedness promote greater autonomous motivation which corresponds to more positive outcomes. The need for competence is satisfied when individuals feel efficacious and perceive they can meet the demands placed upon them. Satisfaction of the need for autonomy entails that individuals feel they have choice and input and act out of personal volition. The need for relatedness is fulfilled when we are connected with others in a caring and supportive manner. Basic Needs Theory assumes that need satisfaction is fundamental for autonomous motivation and, in turn, optimal functioning, personal growth and well-being [14].

Evidence exists regarding the relevance of autonomous motivation and need satisfaction for participation in physical activity [15,16] as well as positive health behaviour change and maintenance in diverse areas [17], such as smoking cessation [18], weight loss [19,20], glycemic control [21,22], and adherence to medical prescriptions [23]. Research support exists as well for the premise that autonomy supportive environments afford greater feelings of competence, autonomy and relatedness which correspond to enhanced autonomous motivation or self determination. In a study of overweight/obese individuals involved in a 3 month exercise referral programme [24], an increase in competence and relatedness need satisfaction over time corresponded to greater adherence. Increases in the overall need satisfaction experienced during the scheme positively predicted self-determined motives for PA engagement. Participants with more autonomous motivation exhibited greater well-being over the course of the programme.

Within SDT and Basic Needs Theory in particular [25], social environmental factors are deemed to be critical to motivational processes and ensuing outcomes. In particular, interactions with significant others marked by high autonomy support are assumed to promote greater need satisfaction and thus positive and healthful striving in individuals. Autonomy supportive leaders, for example, would offer choice in activities, acknowledge participants’ perspectives and seek their input, minimise external rewards, provide meaningful information and rationales for requested or encourage behaviours, support personal choice regarding initiations to change behaviour, and assist in reframing failure attempts.

SDT has begun to lay the bases for the development of interventions to promote PA engagement [25-28]. Exercise class students taught by a teacher with a need supportive style were higher in relatedness and competence need satisfaction as well as positive affect through the end of the 10 week course than those instructed by a teacher with a more traditional style [25]. Attendance was also significantly higher in the intervention arm. An RCT aimed at promoting physical activity within the Canadian primary care system reported that patients who received both an autonomy supportive brief consultation from their GP and 3 months SDT-based counselling on PA adoption from an exercise counsellor perceived greater autonomy support for becoming more active than those who were exposed to brief counselling only [26]. The SDT group also exhibited greater autonomous motivation, self-reported PA engagement at 13 weeks and satisfaction of the competence need though the needs for autonomy and relatedness were not assessed. Results stemming from a path analysis testing of an SDT-grounded process model revealed mid-programme levels of autonomous motivation and perceived competence to predict end-of-programme physical activity levels.

In their PESO trial, Silva and colleagues pulled from SDT to develop a 1-year weight management intervention and contrast its effect with a general health programme [27]. Participants were Portuguese women, between 25 and 50 years of age, who were overweight or obese. At the conclusion of the programme, the women in the intervention group lost more weight and engaged in significantly more PA than their control counterparts. The intervention arm participants also exhibited significantly greater autonomous motivation for PA engagement and perceived their team of care providers to be more autonomy supportive than did the women in the general health programme. In this group of women, autonomous motivation was found to predict weight loss at 3 years following the commencement of the programme [29].

In sum, there are compelling theoretical and empirical reasons for pulling from SDT to develop an intervention to be applied within exercise referral consultations and examine the effectiveness of such an intervention. A major purpose of the present trial was to examine within arm change (baseline to 3 months and to 6 months) and compare the effect (at 6 months) of an exercise referral consultation delivered by SDT-trained HFAs with a standard exercise consultation provided by trained HFAs on participants’ self-reported physical activity, associated health behaviours, physical health, and well-being/quality of life. Our prior hypothesis was that participants in the SDT-arm would exhibit more sustained physical activity and thus would report more activity at the 6-month follow-up. We also expected participants in the SDT-arm to exhibit positive change in well-being and quality of life at 3 and 6 months than what would be observed for those in standard provision exercise on referral and predicted there would be between group differences at 6 months favouring the SDT-arm.

As argued by Michie and Abraham [30], it is important to know *how* theory-based interventions work in impacting behavioural change and associated outcomes. Consonant with their perspective and aligned with Williams and colleagues in their determination of the impact of SDT-based intervention programmes [18], we also tested a process model hypothesising positive relationships between HFA autonomy support, and participants’ need satisfaction and degree of autonomous motivation at the end of the 12 week programme (after considering their degree of autonomous motivation when entering the scheme). Those participants who were more autonomous and experienced greater need satisfaction at the conclusion of the scheme were expected to exhibit lower depression at 6 months. We targeted depressive symptoms as an indicator of mental health at follow-up as mild depression was an inclusion criterion for referral into the exercise programme in question. At 3 months, self-determined motivation and need satisfaction were expected to positively relate to positive intentions for engaging in regular PA over the next 3 months. Intentions for engagement in PA were predicted to be associated with greater PA at 6 months follow up.

## Methods

The methods of this study have been described in detail elsewhere [31]. A briefer outline is provided below.

### Design

The study was a cluster randomised controlled trial with the leisure centres that provided the exercise referral service as the unit of randomisation. All the eligible 13 leisure centres in a large UK city were included. The choice of cluster randomisation was based on the need for the health and fitness advisors providing the intervention to work in a different way (as a consequence of those in the SDT arm having received additional training), which meant that individual randomisation would not have been possible.

### Participants

Participants were those people referred to the exercise referral scheme by their GP or practice nurse, who agreed to be part of the study over an 8 month recruitment period. The exercise referral scheme in the targeted city received approximately 3000 referrals each year. Inclusion criteria for the scheme included two or more risk factors for coronary heart disease; people with chronic medical conditions, such as asthma, bronchitis, diabetes, mild anxiety or depression; people for whom regular activity might delay the onset of osteoporosis, people with borderline hypertension and those perceived by the GP or practice nurse to possess motivation to change. Exclusion criteria included: angina, blood pressure greater than 160/102, poorly controlled diabetes or asthma, myocardial infarction within 6-months, established cerebrovascular disease or severe chronic obstructive pulmonary disease. The city in which the trial took place has a relatively young, ethnically diverse population, with about a third of the population non-white [32] and 16.5% born outside the UK at the 2001 census. Each participant received the intervention consistent with his or her assigned HFA. Consent to follow-up as part of the study was secured by the HFA.

### Measures

#### Primary outcome measure

The primary outcome was self-reported physical activity using the 7-Day Physical Activity Recall (PAR) [33]. Time spent in vigorous and moderate intensity physical activity was calculated for all participants at 3 time points (baseline, 3-months and 6-months). When the data were examined, we considered it likely that the data for minutes of moderate intensity walking had been over-reported [34]. Thus a second measure of physical activity, minutes of moderate or vigorous physical activity excluding walking, was calculated.

#### Secondary outcome measures

Physical health outcomes were measured at baseline and 6-months only: body mass index (BMI) and blood pressure (BP), assessed according to British Hypertension Society guidelines [35].

Health status was assessed at baseline, 3 and 6 months via the General Health, Change in Health, and Physical Fitness scales of the Dartmouth CO-OP Charts [36,37].

Mental/emotional well-being and QOL indicators were measured at the 3 time points. Anxiety and depression were assessed via the 14 item Hospital Anxiety and Depression Scale HADS; [38], and feelings of personal energy and vitality using an abbreviated (6 item) version of the Subjective Vitality Scale SVS; [39,40]. Across the 3 assessment points and as indicated via the calculation of Cronbach’s alpha, the Anxiety and Depression subscales of the HADS (.84 - .87 and .80 - .85, respectively) and the SVS (.92 - .95) were found to have high internal reliability. We also administered other scales embedded in the Dartmouth CO-OP Charts [36] to assess negative feelings, difficulty in doing daily activities, and quality of life.

#### Motivation-related processes of change measures

Participants’ perceptions of the degree of autonomy support provided from their advisor were assessed via the 6 item Health Care Climate Questionnaire HCCQ; [41]. An exemplar item would be “I feel that my important other (s) has/ have provided me with choices and options about physical activity and health.” At baseline and 3 months, the items of the HCCQ were marked by high internal consistency (alpha = .95 - .97).

We assessed participants’ reported satisfaction of the three basic needs with respect to their physical activity engagement via the 18 – item Psychological Need Satisfaction in Exercise Scale PNSES; [42]. This multi-dimensional and SDT-grounded measure assesses the degree to which individuals feel competent (“I feel capable of doing challenging exercises”), a sense of autonomy (“I have a say in choosing exercises”) and relatedness (“I feel close to my exercise compatriots”). At 3 and 6 months, the competence, autonomy and relatedness subscales of the PNSES were internally reliable (alpha = .91 - .92, .88 - .91, and .91 - .92, respectively).

Intentions to engage in PA were assessed via 3 items previously used by Edmunds and colleagues in their study of exercise on referral participants [24]. An exemplary item would be “I plan to regularly engage in physical activity (i.e., at least 5 days per week for a total of 30 minutes each day) during the next 3 months.” In the present study, we centred on participants’ responses to this scale at 3 months (at programme end) and our measure of intentions had high internal reliability (alpha = .95).

The 19-item Behavioural Regulations in Exercise Questionnaire-2 BREQ-2; [43] was employed to measure participants’ motivational regulations for exercise engagement. The BREQ-2 taps intrinsic (“I exercise because it is fun”), identified (“It’s important to me to exercise regularly”), introjected (“I feel guilty when I don’t exercise”) and extrinsic (“I exercise because other people say I should”) reasons for participation in physical activity as well as amotivation (“I don’t see why I should have to exercise). All of these subscales, at baseline and 3 months, were found to be internally consistent (alpha = .72 - .92).

### Procedure

All procedures were approved by the School of Sport and Exercise Sciences Ethical Review Committee at the University of Birmingham, UK.

#### Pre-intervention assessments

After informed consent was taken by the HFA, the baseline measures were administered.

#### Randomisation

All 13 leisure centres that provided the exercise referral service in the three Primary Care Trusts (PCTs) in the targeted UK city took part; 6 of these were randomised to the SDT arm and 7 were randomised to current practice. Randomisation was stratified by PCT and deprivation of population served and undertaken by an independent statistician. The leisure centres each had one HFA working in them, apart from one intervention site, which had two.

#### Interventions

##### SDT-based intervention

The HFAs providing the SDT-based intervention attended group and one-to-one training introducing the theory and highlighting related research. The HFAs were also introduced to major principles of and strategies embedded in an autonomy supportive approach to PA promotion and viewed examples of consultations underpinned by SDT [31].

The training pulled from the autonomy supportive protocol for health counselors developed by Williams and colleagues [18]. Discussions during the consultations revolved around the integration of PA with life values. The HFAs were encouraged to use motivational interviewing techniques such as careful listening, parroting/ paraphrasing, handling resistance, and double sided reflection. The SDT-based strategies also included failure normalization and recalibration of implementation plans (HFA and participant working together). The HFAs were requested to target the participants’ feelings during PA in their discussion and provide support of the participants’ internalisation of PA involvement.

The initial consultation revolved around a discussion of the participant’s exercise history and the benefits and risks of increased physical activity (individualised to the participant’s views about the consequences of regular physical activity and personal health risks). Participants’ perspectives regarding the advantages and disadvantages of change regarding physical activity levels and the perceived barriers to and resources for change were solicited. The HFA also was requested to encourage participants to consider how their intention(s) to become more active might be implemented, and where and how they could secure offerings of social support regarding exercise engagement. Drawing from the information on physical activity participation provided by the 7 day PAR, the consultation concluded with the HFA and participants engaging in specific goal setting for PA participation in the subsequent week. The participants were then offered a fitness assessment (consistent with the standard exercise referral scheme). They were also given a self-management exercise promotion booklet designed to encourage a more autonomous perspective on physical activity initiation. This booklet was developed from existing and successful physical activity promotion materials in the literature (e.g. the “Walk in to Work Out” pack [44] and the Diabetes Prevention Program’s Lifestyle Change Program Manual [45] but worded in a way that it was consonant with the tenets of Self determination Theory.

The intervention also was to include further brief interactions between the participants and the HFA (by telephone or in person) at 1 and 2 months with a focus on sustaining any positive changes made, re-framing and problem solving where attempts to be physically active were not successful, addressing barriers to activity, and setting new personal PA goals. The intervention also entailed a final consultation at 3 months focused on recognising and facilitating the internalisation of the participant’s physical activity involvement, feelings about engaging in physical activity, and planning for future maintenance of activity. A self-management booklet given at the conclusion of the exercise on referral programme centred on the monitoring and maintenance of physical activity. More detail on the SDT-grounded intervention can be found in the detailed protocol [31].

##### Standard provision

After referral by their general practice, participants receiving the standard exercise referral provision had a one hour consultation at their local leisure centre. During this consultation, the HFA asked the client about his/her current state of health, medical problems, medications taken and current physical activity levels using the 7-day PAR. The HFA then described the range of activities available to the participant, both within the leisure centre and in the community. The HFA and participant negotiated and agreed an appropriate programme of individual and/or group activities to help the participant achieve their desired outcome. Participants also had the offer of a fitness assessment, which was not commonly taken up. Over the following 10–12 weeks, the clients undertook their exercise programme with support provided by the HFA as required. At the end of the programme, the HFA invited the patient to an exit consultation to discuss future participation in physical activity. If the participant did not take up the possibility of a one to one exit consultation, a telephone consultation was offered.

#### Follow-up assessments

The primary outcome measure at 3 and 6-months follow-up (i.e., the 7 day PAR) was administered over the telephone by a trained research assistant to ensure blinding, as due to the cluster nature of the trial, it was not possible for the face-to-face follow-up assessments to be blinded. At 3- and at 6-months, the follow-up assessments were undertaken by a member of the research team, not the HFA who had delivered the intervention.

#### Numbers of recruits and sample size calculations

A sample size of 494 participants was required to detect a difference in mean physical activity time across the 2 groups of 100 minutes with 80% power and 0.05 significance level. This estimate is based on a standard deviation of 211 mins [46] and an intracluster correlation coefficient of 0.04 [47]. However, due to difficulties with the recruitment of participants in the early stages of the trial, 347 participants were involved in the trial. This sample size was sufficient to achieve 90% power and 0.05 significance to detect a within group increase of 60 minutes of self-reported physical activity from 108 (sd 211) at baseline.

#### Data analysis

As the physical activity data were strongly skewed, the data were log transformed for the between group analyses. Due to the nested design, the between-group analyses were undertaken using a multilevel modelling approach with MLWin 2.18. Three levels were specified: time (baseline, 3 and 6-months), individuals and leisure centres. First, intraclass correlations were calculated by dividing the between-centres variance by the sum of the variances across the three levels. We then ran a series of linear growth models with arm as a dichotomous predictor and the targeted primary and secondary outcomes as dependent variables. We also tested time X arm intervention effects (in essence, the difference in the baseline to 6 month slope between the two arms). The slope represents the linear change/rate of change in the dependent variable over time. We report whether this linear change is significantly different from zero in the SP group and whether there is a statistically significant difference in the slopes of the SP and the SDT groups.

Where data were missing due to loss to follow-up, data were imputed conservatively using the value at baseline, thus assuming no change in non-respondents (i.e. intention to treat analysis).

## Results

### Recruitment

**Figure 1:**
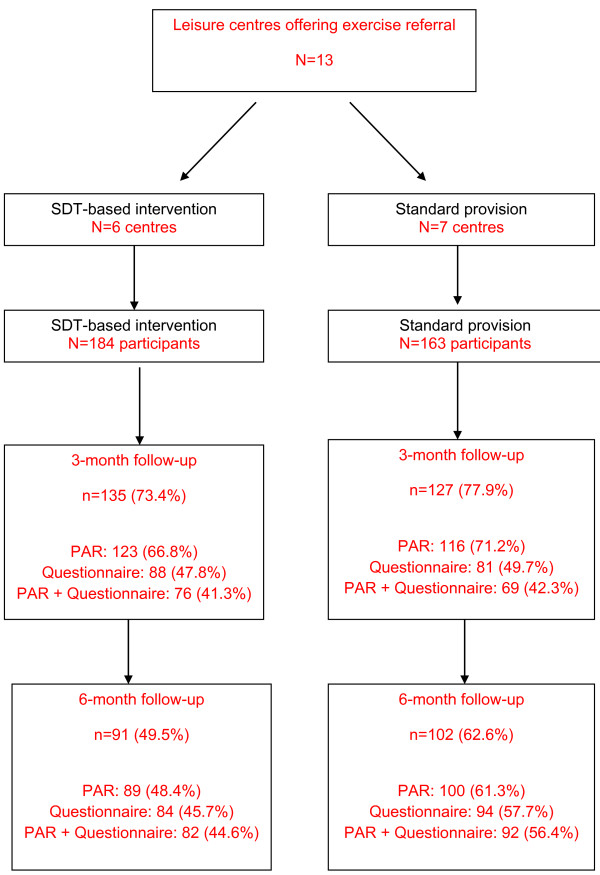
Flow of participants in trial through follow-up.

Of the 1683 people referred to the HFAs during the recruitment period, 347 (20.6%) were recruited and completed the baseline assessment: 184 (53%) were recruited in the SDT-based intervention arm leisure centres and 163 (47%) in leisure centres providing standard provision exercise referral (Figure 1).

### Characteristics of participants

The majority (72.9%) were female and 28.3% were from non-white UK ethnic groups. Overall 72.4% (234) of the participants reported doing less than the Government recommendation of 150 minutes of moderate physical activity, including walking, each week. The majority of participants (90.3%) were either overweight or obese. Using a cut-off of >11 on the Hospital Anxiety and Depression subscales [48], 18.9% of the participants were identified as having probable clinical depression, and 34.8% probable anxiety. Participant characteristics are shown in Table 1.

**Table 1:** Characteristics of the participants

	**SDT-arm**		**Standard provision**	
	**N = 184**	**%**	**N = 163**	**%**
**Age group**				
<30 years	19	10.3	11	6.7
30–49 years	76	41.3	77	47.2
50–64 years	64	34.8	50	30.7
65+ years	25	13.6	25	15.3
**Gender**				
Male	45	24.5	49	30.1
Female	139	75.5	114	69.9
**Ethnic group**				
White British or Irish	134	74.9	104	67.5
Black Caribbean or African	19	10.6	23	14.9
South Asian	17	9.5	23	14.9
Mixed race and others	9	5.0	4	2.6
**Qualifications**				
None or up to GCSE or equivalent	104	64.2	87	58.0
**Referral**				
Initiated by primary care team	64	54.2	58	58.6
Client asked for referral	54	45.8	41	41.4
**Clinical indices**				
**Smoker**	40	22.1	33	23.1
**Hypertensive (>140/90 mmHg)**	70	38.0	60	37.5
**BMI (kg/m**^**2)**^				
Normal (<25)	18	10.3	13	8.3
Overweight (25–29)	44	25.3	41	26.3
Obese (30–39)	91	52.3	81	51.9
Morbidly obese (40+)	21	12.1	21	13.5
**Psychological state**				
Probable anxiety (HADSa 11+)	68	34.2	52	31.9
Probable depression (HADSd 11+)	40	21.9	25	15.3
**Physical activity levels (mins mod or vigorous activity)**				
Sedentary (30 mins or less/week)	75	44.9	71	45.8
Below recommended level (31–149 mins/week)	48	28.7	45	29.0
151–419 mins/week	32	19.2	24	15.5
420 mins/week (>1 hour per day)	12	7.2	15	9.7
**Alcohol intake within previous week (units)**				
None	102	58.3	68	50.7
<21 units	70	40.0	64	47.8
>21 units	3	1.7	2	1.5

#### Follow-up rates

Follow-up rates are shown in Figure 1. Overall follow-up at 3-months was 75.2% and at 6-months follow-up was 55.6%. At 6-months there was a differential follow-up rate between the study arms, with a lower rate of follow-up in the SDT-based arm (p = 0.02).

### Between group comparisons

Between group change over the 6-month period of follow-up was assessed with multi-level modelling as detailed in the methods section. Table 2 presents the ICC for each variable, the mean values of the dependent variables for the standard provision arm at the end of the study (as time was centered at 6 months), the mean differences between the two arms at the end of the study and the statistical significance of such differences. Table 2 also presents the change in the slope from baseline to 6 months for all variables in the standard provision group, the difference in the slope between the two arms, and the statistical significance of such difference.

**Table 2:** Physical and psychological outcomes: multilevel models for standard provision (S-P) and SDT provision (SDT-P)

	**Mean S-P at 6 months**	**Mean difference S-P and SDT-P at 6 months**	**P value for mean difference**	**Slope from baseline to 6 months in the SP group**	**Difference in the slope between S-P and SDT-P**	**P value for slope difference**	**ICC**
Minutes of physical activity/week (ln)^1^	4.35	-.03	.93	.49**	-.09	.50	0.06
Minutes of physical activity minus walking/week (ln)^1^	2.95	.22	.50	.29**	.01	.95	0.09
Vitality^2^	3.79	.23	.17	.14**	.01	.87	0
HADS anxiety score^3^	8.79	1.00	.03	-.22*	.01	.47	0
HADS depression score^3^	6.53	-.56	.22	-.37**	.13	.34	0
Dartmouth quality of life domains^4^							
Physical fitness	2.88	.08	.51	.08*	-.06	.24	0
Emotional problems	3.20	-.06	.64	.10**	-.13	.004	0
Daily activity	3.42	.08	.48	.10**	-.08	.08	0
Change in health	3.21	.09	.27	.03	-.03	.64	0.02
Overall health	2.53	.14	.15	.10**	-.06	.15	0.01
Quality of life	3.17	0.07	.40	.06*	-.01	.08	0
Systolic blood pressure (mmHg)	127.39	1.74	.40	-	-	-	0
Diastolic blood pressure (mmHg)	79.72	0.98	.35	-	-	-	0.01
Weight (kg)	89.36	1.56	.40				0

^1^Activity of at least moderate intensity.

^2^Positive score associated with improved vitality.

^3^Positive score associated with greater psychological morbidity.

^4^Positive score associated with improved quality of life.

Ln: natural log.

*p < .05 **p < .01; Indicate whether the change in the slope from baseline to 6 months in the SP group was significantly different from zero.

As can be seen from Table 2, the ICC for each of the dependent variables was very low. Groups were not significantly different for either moderate/vigorous PA or PA excluding walking. The SDT-based arm had significantly lower anxiety scores over the follow-up period (difference between SDT and standard provision −1.00, p = .003). No significant differences in the slopes between the two arms were found except in the case of the reported emotional problems as assessed via the Dartmouth Charts.

### Within group change

#### Standard provision arm

##### 3-months follow-up

Participants in the standard provision arm exhibited a significant increase of 187 minutes (95% CI 131, 243) of self-reported moderate or vigorous physical activity and 112 minutes (95% CI 62, 162) increase of moderate/vigorous physical activity excluding walking at 3 months. Subjective vitality significantly improved and levels of anxiety and depression reduced significantly. Significant improvement also was observed in the physical health domains of physical fitness, change in health and overall health emerged, as assessed via the Dartmouth Charts (Table 3).

**Table 3:** Within group change from baseline to 3 and 6 months follow-up (BOCF)

	**Baseline to 3-months follow-up**						**Baseline to 6-months follow-up**			
	**SDT-provision**			**Standard provision**			**SDT-provision**		**Standard provision**	
	**Baseline value Mean (sd)**	**Difference**	**95% CI**	**Baseline value Mean (sd)**	**Difference**	**95% CI**	**Difference**	**95% CI**	**Difference**	**95% CI**
Minutes of physical activity/week^1^	132 (237)	196**	144, 248	134 (240)	187**	131, 243	114**	70, 159	120**	67, 172
Minutes of physical activity minus walking/week^1^	81 (192)	110**	71, 148	88 (209)	112**	62, 162	61*	23, 100	73*	18, 128
Vitality^2^	3.34 (1.6)	0.36**	0.22, 0.50	3.63 (1.5)	0.31**	0.15, 0.47	0.34**	0.17, 0.51	0.34**	0.13, 0.55
HADS anxiety score^3^	9.30 (4.4)	−0.41*	−0.70, -0.12	8.14 (4.5)	−0.41*	−0.78, -0.04	−0.44*	−0.80, -0.08	−0.24	−0.63, 0.16
HADS depression score^3^	7.38 (3.91)	−0.70**	−0.97, -0.42	6.58 (4.0)	−0.64*	−1.03, -0.25	−0.73**	−1.07, -0.03	−0.47*	−0.90, -0.04
Dartmouth quality of life domains^4^										
Physical fitness	2.68 (1.1)	0.20*	0.07 0.32	2.91 (1.2)	0.11*	0.01, 0.22	0.15*	0.01, 0.30	0.02	−0.13, 0.17
Emotional problems	2.96 (2.96)	0.16	0.08, 0.5	3.19 (1.2)	0.01	−0.12, 0.11	0.19*	0.07, 0.32	−0.07	−0.23, 0.09
Daily activity	3.18 (1.0)	0.15	0.02, 0.27	3.45 (1.0)	0.04	−0.06, 0.15	0.20**	0.08, 0.32	0.06	−0.08, 0.20
Change in health	3.10 (0.8)	0.14*	0.04, 0.22	3.27 (0.7)	0.10*	0.01, 0.21	0.06	0.03, 0.16	−0.01	−0.13, 0.12
Overall health	2.29 (0.9)	0.19*	0.08, 0.30	2.58 (0.9)	0.12*	0.03, 0.20	0.21**	0.12, 0.30	0.06	−0.08, 0.20
Quality of life	3.02 (0.8)	0.14	0.0, 0.22	3.25 (0.8)	0.01	−0.08, 0.06	0.12*	0.04, 0.21	−0.01	−0.12, 0.11
Systolic blood pressure (mmHg)	129.3 (13.9)	-	-	133.6 (14.8)	-	-	−2.84	−6.57, 0.82	−3.53	−7.31, 0.25
Diastolic blood pressure (mmHg)	78.6 (10.0)	-	-	80.5 (9.3)	-	-	0.77	−2.07, 3.61	1.55	−1.02, 4.11
Weight (kg)	89.3 (18.8)	-	-	91.9 (22.4)	-	-	−0.14	−0.52, 0.22	−0.77*	−0.38, -0.16
Body mass index (kg/m^2^)	32.8 (6.3)	-	-	33.1 (6.9)	-	-	−0.07	−0.21, 0.07	−0.24*	−0.45, -0.03

^1^Activity of at least moderate intensity.

^2^Positive score associated with improved vitality.

^3^Positive score associated with greater psychological morbidity.

^4^Positive score associated with improved quality of life.

*p ≤ 0.05; **p ≤ 0.001.

*BOCF*: baseline observation carried forward.

##### 6-months follow-up

From baseline to 6-months, the increase in reported moderate or vigorous physical activity from baseline was 120 minutes (95% CI 67, 172) and for moderate/vigorous physical activity excluding walking it was 73 minutes (95% CI 18, 128), both statistically significant. There was a significant improvement in reported feelings of vitality and a significant reduction in the HADS depression scores, but no significant change in anxiety scores. No significant improvement was seen for any of the Dartmouth quality of life domains (Table 3). There were also no significant changes in systolic or diastolic blood pressure but weight and BMI reduced significantly from baseline to 6 month follow up, although by a clinically small amount (Table 3).

#### SDT-based intervention group

##### 3-month follow-up

In the SDT-based intervention arm, there were significant improvements from baseline to 3 month follow-up in moderate to vigorous physical activity (and moderate to vigorous physical activity excluding walking), feelings of vitality, scores on the HADS anxiety and depression scales, and the Dartmouth domains of physical fitness, change in health and overall health improved significantly (Table 3). Moderate/vigorous physical activity increased by 196 minutes (95% CI 114, 248) and physical activity excluding walking increased by 110 minutes (95% CI 71, 148) from baseline.

##### 6-month follow-up

In the intervention arm, participants self-reported moderate/vigorous physical activity increased by 114 minutes from baseline (95% CI 70, 159) and physical activity excluding walking increased by 61 minutes (95% CI 23, 100). Vitality and HADS anxiety and depression improved significantly when compared to baseline. Significant improvements from baseline to 6 months occurred in all the Dartmouth quality of life domains, except for reported change in health (Table 3). There were no significant differences from baseline to 6-months in BP, BMI, or weight for participants in the intervention arm.

### Test of the hypothesised process model

In line with previous work by Williams and associates [18,21], the process model for the effects of perceived HFA autonomy support, need satisfaction, and self determination for exercise engagement on participants’ follow-up physical activity and mental health was tested on the combined data from the two arms. Structural equation modeling (SEM) was conducted using EQS 6.1 [46] to assess whether the hypothesized model was supported by the data. A robust maximum likelihood estimation method of analysis was implemented and a number of fit indices were examined to assess model fit. These were the Bentler-Bonnet Non-Normed Fit Index (NNFI), the Comparative Fit Index (CFI), the Standardized Root Mean Square Residual (SRMR), and the Root Mean Square Error of Approximation (RMSEA) with its 90% confidence interval (CI). Although universally accepted cut-off values for the different indices of model fit do not exist, Hu and Bentler recommended that NNFI and CFI values that are equal or above .95, a RMSEA that is equal to or less than .06 and a SRMR that is equal to or less than .08 indicates a model with good fit to the data [47].

We did not model all individual items from all scales in order to increase the stability of parameter estimates and keep an acceptable ratio of sample size to estimated parameters in studies involving relatively low sample sizes [48]. We used parcels which are aggregate indicators created by averaging two or more items from a questionnaire. According to Marsh and colleagues [49], parcels have the advantage of being more reliable and more normally distributed than individual items. Four parcels were created for the self determination (RAI) index each formed by averaging 5 items representing the different motivational regulations for participating in physical activity (i.e., intrinsic, identified, introjected, and external regulations). Following the procedure employed by Markland and Ingledew [50], we calculated the RAI by differentially weighting each subscale and summing the weighted scores such that the final index represents the overall degree of relative autonomy in the regulation of exercise behaviour. The parcels were then formed based on the factor loadings (i.e., the largest factors were paired with the smallest factors).

The three parcels for need satisfaction represented averaged item scores for autonomy, competence and relatedness (3 months). Three parcels for health and fitness advisor autonomy support (3 months) and depressive symptoms (6 months) were created based on the factor loadings (i.e., the largest factors were paired with the smallest factors). Table 4 provides the factor loadings of the parcel indicators for each latent variable. No parcels were created for physical activity intentions as these were measured with three items serving as indicators of the latent variable. Physical activity (6 months) was an observed variable and had no indicators.

**Table 4:** Loadings and residual variances of the parcels and indicators for the latent variables in the structural model

**Psychological variable**	**Parcel/indicator**	**Standardized loading**	**Uniqueness**
Self determination (Baseline)	Parcel 1	.87	.50
	Parcel 2	.84	.55
	Parcel 3	.77	.64
	Parcel 4	.80	.60
Self Determination (3 months)	Parcel 1	.79	.62
	Parcel 2	.84	.54
	Parcel 3	.90	.44
	Parcel 4	.80	.60
HFA autonomy support (3 months)	Parcel 1	.94	.35
	Parcel 2	.92	.40
	Parcel 3	.97	.24
Need satisfaction (3 months)	Parcel 1	.72	.70
	Parcel 2	.71	.71
	Parcel 3	.65	.77
Physical activity intentions (3 months)	Indicator 1	.93	.37
	Indicator 2	.95	.31
	Indicator 3	.86	.52
Depression (6 months)	Parcel 1	.79	.61
	Parcel 2	.80	.60
	Parcel 3	.84	.54

Testing of the original hypothesized model revealed that some indices indicated an adequate fit to the data [CFI = 0.98; NNFI = 0.96, RMSEA = 0.05 (90% CI = 0.03-0.06)], however, the SRMR was relatively high (0.11). Therefore, the Lagrange Multiplier (LM) test was employed to investigate misspecifications in the hypothesized model. The LM modification indices highlighted that the addition of a path between participants’ degree of self determination at baseline and need satisfaction at 3 months would improve model fit. This modification was implemented.

**Figure 2:**
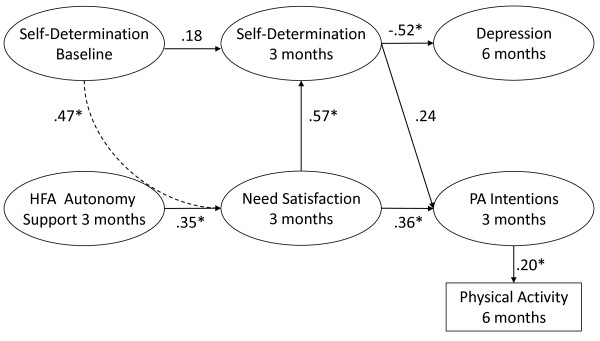
Process model predicting physical activity and mental health (depression) at follow-up.

Figure 2 displays the re-specified model which showed a good fit to the data: CFI = 0.98; NNFI = 0.98, RMSEA = 0.04 (90% CI = 0.02-0.06); SRMR = 0.06. The re-specified model indicates that baseline self determination did not predict the corresponding degree of self determination (RAI index) at 3 months. However, the participants’ degree of self determination at baseline was a positive predictor of need satisfaction at programme end. Perceptions of autonomy support provided by the health and fitness advisor at 3 months were positively linked to participants’ psychological need satisfaction at the conclusion of the 3 month scheme. The latter variable positively predicted changes in self determination from baseline to 3 months. Further, physical activity intentions were positively predicted by psychological need satisfaction at 3 months, but not by changes in the degree of self determination for engagement in physical activity. Finally, physical activity intentions at 3 months positively predicted physical activity behavior at 6 months and changes in the RAI were negatively associated with depressive symptoms (6 months).

We ran an additional model with “arm” predicting all 4 motivation-related process variables in the model, plus intentions to engage in regular physical activity post-programme and reported physical activity and depression at 6 months. This model had very similar fit to the modified model described above and the path coefficients associated with “arm” were non-significant. These findings provide an empirical justification for collapsing participants across arms in our test of the hypothesised process model.

## Discussion

There have been consistent calls for more theory-based research in the area of health promotion [9,10]. This literature also points to the importance of testing the hypothesised processes by which theory-informed interventions are expected to impact targeted outcomes [11]. This trial examined the between arm effect (at 6 month follow-up) of a Self Determination Theory grounded [12,13] exercise referral consultation with a standard exercise consultation on participants’ self-reported physical activity, associated health behaviours, physical health, and well-being/quality of life. Within-arm changes in the targeted outcomes (baseline to 3 as well as 6 months) were also examined. We also tested a process model depicting expected relationships between the degree of autonomy support deemed to be provided by the Health and Fitness Advisor, changes in participants’ motivational processes, and self-reported physical activity and mental health at follow-up.

Both the standard provision and the SDT-based exercise referral programme achieved significant improvements in self-reported physical activity by the end of the 10–12 week programme, which were largely sustained to 6-months and were of an order that would improve health [51]. These findings are aligned with previous evaluations of exercise referral schemes [1-3] but do provide some evidence for a significant impact in physical activity levels at 6 month follow-up.

We had hypothesised that the SDT-based intervention would sustain the increase in physical activity better than the standard provision programme. However, no difference in physical activity outcomes was observed between the study arms at 6 months. This finding is consonant with other trials that have compared two active interventions [5,6] and reported no significant differences in physical activity between the intervention groups at follow-up.

In a validation sub-study [52], we video-recorded a sampling of consultations and objectively rated them for autonomy support, need support and structure. Whilst overall need support was higher in the consultations of the SDT-trained HFAs, the specific provision of autonomy support was not. It could have been the case that some of the standard provision HFAs were naturally working in an autonomy supportive manner or the training offered to the intervention HFAs was not sufficient to alter this dimension of the consultation experience. Consistent with these suggestions, there were no differences between the arms in perceived autonomy support by the HFA. Striking ceiling effects in scores on the Health Care Climate Questionnaire were observed in this study which also could have contributed to the insignificant effect of arm on perceptions of HFA autonomy support [28].

This was an exploratory trial and it is important to note the challenges in implementing the intervention. We met with considerable obstacles in training and supporting the HFAs who were assigned to the SDT-based arm. Opportunities for training days were limited and the HFAs were also taking external examinations to comply with recent guidance for exercise referral professionals, which occurred in the same period as our training. These additional work-related demands may have reduced the importance and/or attention given by the HFAs to the SDT-based training. Additionally, several HFAs worked with limited access to email or computers, so receiving reminders from the research team and watching training videos proved difficult. Such factors may have resulted in our intervention having been less completely and rigorously implemented than we planned. Future work testing SDT-based interventions in physical activity promotion should aim to overcome these challenges in implementation and thus allow a more bonafide examination of intervention efficacy.

In contrast to our findings, Fortier and colleagues reported greater self-reported PA engagement at 13 weeks following an autonomy supportive consultation provided by a physical activity counsellor with brief consultation by the GP within primary care compared to brief counselling only [26]. Silva and colleagues [27], in their SDT-grounded intervention focused on overweight and mildly obese women, reported significantly greater engagement in moderate-vigorous physical activity at the end of the 1 year programme (ES = 1.14) but also at the 2 year follow up. It should be noted, however, that the Fortier et al. intervention and particularly the PESO trial were more intensive than the present intervention. The former entailed approximately 280 minutes of contact while the latter involved approximately 30 groups sessions over the 1 year intervention. It could be argued that such intense interventions are not pragmatic within the constraints of the UK National Health Service. Further, the Fortier intervention compared an intensive intervention by an exercise counsellor plus brief physician advice to brief physician advice alone.

SDT assumes that environments that support basic need satisfaction should lead to not only behavioural persistence but also optimal functioning as reflected in decreased ill-being and enhanced well-being [14]. In both arms, feelings of vitality and psychological health were improved at the end of the 3 month programme. Both interventions also led to enhancements in self-reported physical fitness, change in health and overall health after 3 months. Within-arm analyses however revealed all indicators of quality of life as tapped via the Dartmouth Charts except one (i.e., change in health) to be significantly enhanced at 6 month follow-up, when compared to baseline values, only in the SDT-based arm. Although both arms exhibited positive and significant baseline to 6 month change in feelings of vitality, only in the SDT-base arm did the observed decreases in reported anxiety reach statistical significance.

Consonant with theoretical predictions and suggesting the motivation to engage in physical activity was more emotional integrated, between arm analyses revealed participants in the intervention arm to report significant improvements in experienced anxiety symptoms at 6-months beyond those seen in the standard provision arm. Research has indicated that negative emotional states are predictive of decreases in subsequent levels of physical activity [53]. Thus, it would have been interesting to examine whether the improved mental health observed for intervention participants at 6 months (when contrasted to standard provision controls) would have translated into significantly greater physical activity engagement at 9 and 12 months and beyond.

The results of the process model were also aligned with theoretical predictions. The findings suggest that the level of autonomy support provided in the exercise on referral service and related changes in motivational processes in the participants were predictive of enhanced mental health (i.e., lower depressive symptoms) and reported physical activity at follow-up.

### Strengths and limitations

Our follow-up rates were 75.2% at the 3-month and 55.6% at the 6-month follow-up. This is in keeping with the follow-up rates of several other trials of exercise referral programmes [6,54,55]. To ensure that we did not over-estimate the public health impact of the interventions, we used the baseline observation carried forward for all missing data in the analyses. Thus, analyses were all by intention to treat.

We did not manage to recruit the number of participants required from our power calculation and, thus, the lack of further differences between study groups may be a result of an underpowered study. We did, however, have adequate power for the within group analyses for change over time. Recruitment was undertaken by the exercise referral staff and thus may have led to some recruitment bias, given that they could not be blinded to the study arm. Low recruitment rates were in part due to the ethnic diversity of the population studied and difficulties with administering the study questionnaires to people who did not speak English with sufficient fluency. The use of interpreters was not easy to organise to coincide with a convenient time for both the client and interpreter. Therefore, the participants recruited were all adequate speakers of English and not fully representative of the local population. Follow-up was blinded and undertaken by the research team.

Although reflective of the norm to date in trials assessing the effectiveness of exercise on referral schemes [2], a limitation of this study is that physical activity behaviour was assessed via self-report. Future work examining the impact of such a theoretically-grounded intervention within exercise on referral would be strengthened via the use of objective measures of physical activity.

## Conclusions

The present trial is one of the first to examine the effectiveness of a SDT-grounded physical activity consultation and entails the first to test this intervention approach within an exercise referral scheme. Between arm comparisons indicated the intervention arm to result in greater reductions in reported anxiety at 6 months. The findings suggest that both standard provision and an SDT-based exercise referral programme impacted self-reported physical activity levels and most of the targeted indicators of mental health to 6 month follow-up. Via the testing of a process model, evidence was accrued for the relevance of need supportive consultations to corresponding changes in participants’ basic need satisfaction and motivation for engagement. These motivational processes were predictive of participants’ emotional well-being and levels of moderate-vigorous physical activity post-programme.

## Competing interests

The authors declare they have no competing interests.

## Authors’ contributions

JD and KJ led on the project and JD, KJ, NN, AD, FE, NM and GW participated in study design and wrote the initial protocol. GW, JD, KJ, FE, NM and RL delivered elements of the training programme. RL, PR and RB co-ordinated the study with supervision from JD and KJ, KJ, NN and PR undertook the analysis. JD and KJ drafted the manuscript. All authors read and approved the final manuscript.
